# Health-Related Quality of Life Outcomes With Regular Yoga and Heartfulness Meditation Practice: Results From a Multinational, Cross-sectional Study

**DOI:** 10.2196/37876

**Published:** 2022-05-17

**Authors:** Jayaram Thimmapuram, Kamlesh Patel, Divya K Madhusudhan, Snehal Deshpande, Ekta Bouderlique, Veronique Nicolai, Raghavendra Rao

**Affiliations:** 1 Internal Medicine Department Wellspan York Hospital York, PA United States; 2 Heartfulness Institute Hyderabad India; 3 Global Clinical Scholars Research Training Department of Medical Education Harvard Medical School Boston, MA United States; 4 Sneh Rehabilitation Education and Research Center Mumbai India; 5 Central Council for Research in Yoga and Naturopathy New Delhi India

**Keywords:** yoga, meditation, health-related quality of life, Heartfulness, COVID-19, healthy living, wellness, quality of life, stress, mental health, psychological health, online survey, cross-sectional study, health outcome

## Abstract

**Background:**

Although the benefits of yoga are well established across the world, there are limited studies exploring the long-term interrelation between yoga, meditation, and health. Specifically, there is limited research exploring the differences in health-related quality of life (HRQOL) among regular meditators and nonmeditators.

**Objective:**

This study explored the differences in 7 domains of HRQOL (including quality of life, ability to adopt a healthy lifestyle, ability to relax, frequency of nervousness and stress, coping with day-to-day stress, workplace productivity, and staying healthy during the COVID-19 pandemic) among practitioners of yoga and meditation.

**Methods:**

A cross-sectional, online survey was distributed to all members who participated in a 100-day yoga and meditation program, culminating in the International Day of Yoga event, organized by the Heartfulness Institute in partnership with the Central Council for Research in Yoga and Naturopathy, Ministry of Ayush, SVYASA Yoga University, and Patanjali Yoga Institute, India. The program consisted of daily virtual yoga, meditation, and speaker sessions. The data were analyzed by nonparametric Mann-Whitney *U* test and Kruskal-Wallis tests for continuous variables and chi-square test for categorical variables.

**Results:**

A total of 3164 participants from 39 countries completed the survey. Mean age was 33.8 (SD 13.6) years. The majority of the participants were female (n=1643, 52%) and students (n=1312, 41.5%). Regular yoga and meditation practice was associated with a positive impact on all 7 domains of HRQOL (Mann-Whitney *P*<.05 and χ^2^ *P*<.05). Notably, experienced Heartfulness (≥2 years) meditators reported better outcomes in all the domains of HRQOL as compared to those not currently practicing this form of meditation and participants with ≤1 year of Heartfulness meditation experience (*P*<.05).

**Conclusions:**

This is one of the first cross-sectional studies to explore HRQOL outcomes among participants of a 100-day virtual yoga and meditation program. Overall, a yoga and meditation practice was found to be an effective tool for promoting HRQOL. Regular yoga and meditation practice was associated with factors promoting health and well-being, with long-term meditation practice associated with increased benefits.

## Introduction

The COVID-19 pandemic is an unprecedented crisis, the effects of which have been felt globally [1-3]. In March 2020, there were 372,757 reported cases from 170 countries, followed by a rapid rise in cases and geographical spread, with over 440 million people affected by COVID-19 globally as of February 2022; during the past 2 years, the pandemic caused disruptions in physical, mental, and emotional health, severely impacting health-related quality of life (HRQOL) [4-6]. HRQOL is an individual’s or a group’s perceived physical and mental health over time [7]. It is an important measure used to assess the impact of diseases or disabilities on the physical, mental, and social domains of population health [8]. A growing body of evidence suggests the current pandemic has had a substantial negative impact on various dimensions of HRQOL, thus highlighting the need to prioritize both mental and physical health dimensions in these challenging times [9-11].

Prior literature has suggested the practice of yoga and meditation can significantly improve an individual’s HRQOL [12-14]. Yoga, a mind-body practice that includes a combination of physical poses, regulated breathing, and meditation, is one of the world’s most popular practices for general well-being [15]. Yoga and meditative practices are an effective intervention for chronic health conditions including diabetes, cardiovascular disease, metabolic syndrome, and cancer [16,17]. Furthermore, the practice is beneficial in decreasing inflammation and improving immune system function, favorably affecting mental health by reducing depression and anxiety [18-24]. Although the benefits of yoga and meditation are well established around the world, there are limited studies exploring the long-term interrelation between yoga, meditation, and health [14]. Specifically, there is limited research exploring the differences in HRQOL among meditators and nonmeditators. The aim of this study was to explore the differences in 7 domains of HRQOL (quality of life, ability to adopt a healthy lifestyle, ability to relax, frequency of nervousness and stress, coping with day-to-day stress, workplace productivity, and staying healthy during the COVID-19 pandemic) among individuals who participated in a 100-day virtual yoga and meditation program, culminating in the International Day of Yoga event.

## Methods

### Study Design

This is a cross-sectional study that included participants aged ≥18 years from 39 participating countries. The online survey was administered to all participants registered for the International Day of Yoga event, organized by the Heartfulness Institute in partnership with the Central Council for Research in Yoga and Naturopathy (CCRYN), Ministry of Ayush, SVYASA Yoga University, and Patanjali Institute, India. Individuals included in the study (1) were at least 18 years of age, (2) had internet access and the ability to complete an online survey either in English or Hindi, and (3) had registered to participate in a 100-day virtual yoga and meditation program. Participants for the event were recruited by multiple channels including social media, partner organizations, and word of mouth. All participants completing the survey and consenting to the use of data for research purposes were included in the analysis. Ultimately, 3164 participants were included in the analysis.

### Intervention

The virtual 100-day yoga and meditation program ran from March 14, 2021, through June 21, 2021. The event was telecasted on YouTube and social media pages including Facebook. The program was facilitated by certified yoga and Heartfulness meditation trainers and consisted of live yoga asanas (postures) in the tradition of Ashtanga yoga, meditation, and speaker sessions. Asana sessions consisted of breathing exercises (pranayama), sun salutation (surya namaskar), yoga practice (beginner, intermediate, and advanced levels as the program progressed), and meditation sessions. Meditation sessions were based on Heartfulness practices. Participants were requested to sit comfortably with eyes closed and gently focus their attention on the source of light within their heart. Participants were asked to simply tune into their hearts and be open to any experience they may have as opposed to trying to visualize the light. If their attention drifted, participants were advised to gently redirect their attention toward their heart. This form of meditation practice has been studied in multiple settings, demonstrating favorable outcomes on burnout, sleep, loneliness, heart rate variability, and emotional well-being [25-33]. Further, speaker sessions included subject matter experts on yoga and meditation from around the world who spoke about topics including but not limited to history of yoga, yoga for unity and well-being, effect of yoga on different systems in the body, benefits of meditation, and research in yoga. The duration of the daily sessions was approximately 1 hour.

### Survey Instrument and Data Collection

The survey was designed using standardized scales for well-being–related measures along with questions on demographics and patterns of yoga and Heartfulness meditation practice and administered as a Google Form. The survey was developed in partnership with the CCRYN, Ministry of Ayush, Government of India, to ensure representation of global yoga and meditation practices. Administered in both English and Hindi languages, the survey consisted of 20 items and was divided into three parts: (1) participant demographics including age, gender, country of residence, and occupation, (2) regularity of yoga practice and duration of meditation practices, and (3) HRQOL questions rated on a scale from 0-10 on quality of life, ability to adopt a healthy lifestyle, coping with day-to-day stress, workplace productivity, and staying healthy during the COVID-19 pandemic. Further, a Likert scale was used to assess the domains of ability to relax and frequency of nervousness and stress. Data were collected over a 2-week period.

### Ethical Considerations

This study was cross-sectional in nature and was conducted as a program evaluation. As such, it was exempt from institutional review board approval. However, informed consent and password protection for data collection were included. This e-survey design was reported using the CHERRIES (Checklist for Reporting Results of Internet E-Surveys) guidelines [34]. Participation was voluntary and included signing an electronic informed consent form prior to accessing the survey questionnaire.

### Cost to Participants

Participants did not incur any costs associated with the event and did not receive any incentives for study participation. All participants received a certificate of participation from the Heartfulness Institute, India, at the end of the program.

### Data Analysis

Survey results were cleaned to identify miscoded, missing data and outliers. Data were entered in Microsoft Excel and analyzed using SPSS (version 22; IBM Corp). Descriptive statistics (frequencies, percentages, and standard deviations) were obtained to describe the demographic data, yoga and meditation practice patterns, and attendance across the 100-day virtual yoga event. Participants were asked to report on the frequency of yoga to examine its relation to HRQOL. The differences in HRQOL among participants who practiced yoga regularly was compared against participants not practicing yoga regularly. Similarly, participants were asked to report on their experience with Heartfulness meditation and duration of practice in years. The differences in HRQOL among participants who practiced Heartfulness meditation regularly (≥2 years) were compared against participants with less experience with this form of meditation (≤1 year) and participants who had previously tried this form of meditation but were not currently practicing. Kolmogorov-Smirnov normality test showed that most data were not normally distributed. Thus, Mann-Whitney *U* test and Kruskal-Wallis tests were used for continuous variables and chi-square test was used for categorical variables. Further, a post hoc analysis of continuous variables was conducted to examine differences in HRQOL within the Heartfulness meditation practitioner group. A *P* value of <.05 was considered statistically significant throughout the analysis.

## Results

### Overview

Participants’ demographic characteristics are described in Table 1. Of the 3164 participants included in the analysis, the majority were female (n=1643, 51.93%) and students (n=1312, 41.47%). Participants’ mean age was 33.87 (SD 13.61, range 18-80) years. Of the 39 countries that participated in the program, most of the participation was from India (n=3020, 95.45%), followed by the United States (n=29, 0.92%) and United Arab Emirates (n=17, 0.53%). Of 3164 participants in the sample, 1647 (52.05%) were regular yoga practitioners, and 1517 were categorized as nonregular yoga practitioners (47.95%). Further, 64.89% (n=2053) reported experience with Heartfulness meditation practice and 35.11% (n=1111) did not practice Heartfulness meditation. Among the Heartfulness meditation practitioners, 38.28% (n=786) reported ≤1 year of practice, 59.28% (n=1217) had practiced for ≥2 years, and 2.44% (n=50) of participants reported not currently practicing this form of meditation.

**Table 1:** Population demographics (N=3164).

Characteristics			Values
**Gender, n (%)**			
	Male	1520 (48.04)	
	Female	1643 (51.93)	
	Other	1 (0.03)	
**Age (years), mean (SD)**			33.87 (SD 13.61)
	Heartfulness meditation group	34.53 (SD 14.46)	
	Non–Heartfulness meditation group	30.41 (SD 12.27)	
**Frequency of yoga practice, n (%)**			
	Regular yoga practitioner	1647 (52.05)	
	Nonregular yoga practitioner	1517 (47.95)	
**Meditation group, n (%)**			
	Heartfulness meditation group	2053 (64.89)	
	Non-Heartfulness meditation group	1111 (35.11)	
**Years with Heartfulness meditation group, n (%)**			
	≤1 year	786 (38.28)	
	≥2 years	1217 (59.28)	
	Not currently practicing	50 (2.44)	
**Occupation, n (%)**			
	Student	1312 (41.47)	
	Government and public sector services	404 (12.77)	
	Professionals (engineers, legal, human resources, etc)	400 (12.64)	
	Others	360 (11.38)	
	Health care professionals	243 (7.68)	
	Homemaker	211 (6.67)	
	Self-employed, entrepreneurs, business	206 (6.51)	
	Farmer	15 (0.47)	
	Armed forces	13 (0.41)	
**Country, n (%)**			
	India	3020 (95.45)	
	United States	29 (0.92)	
	United Arab Emirates	17 (0.54)	
	Canada	10 (0.32)	
	United Kingdom	9 (0.28)	
	Malaysia, France, Oman	8 (0.25) from each country	
	Ukraine, Mauritius	5 (0.16) from each country	
	Brazil	4 (0.13)	
	Australia, Germany	3 (0.09) from each country	
	Austria, China, Indonesia, Iran, Ireland, Italy, Kuwait, Qatar, Uzbekistan	2 (0.06) from each country	
	Argentina, Bahrain, Belarus, Bhutan, Denmark, Hong Kong, Japan, Kenya, Nepal, Mexico, Panama, Philippines, Portugal, Romania, Russia, Spain, Sri Lanka, Venezuela	1 (0.03) from each country	

### Program Engagement

A total of 3164 individuals completed the survey and reported an average participation rate of 71 (SD 32) days. Most participants (n=1684, 53.22%) attended daily yoga and meditation sessions throughout the 100 days.

### Effect of Yoga on HRQOL

Participants who practiced yoga regularly reported a statistically significantly higher positive impact on all domains of HRQOL as compared to participants who were not regular yoga practitioners, including quality of life (*U*=924263.5, *P*<.001), ability to adopt healthy lifestyle (*U*=915778.500, *P*<.001), coping with day-to-day stress (*U*=898958.000, *P*<.001), improving work productivity (*U*=908140.500, *P*<.001), and staying healthy during the COVID-19 pandemic (*U*=896486.500, *P<*.001; Table 2). Further, regular yoga practitioners had a greater ability to relax (*df*=3, *P*<.001) and experienced lower frequency of nervousness and stress (*df*=3, *P*<.001) as compared to nonregular yoga practitioners (Table 3).

**Table 2:** Effect of yoga on health-related quality of life.

Health-related quality of life characteristic	Mann-Whitney *U* test	Wilcoxon *W*	*Z* statistic	*P* value
Quality of life	924263.500	2075666.500	–13.381	<.001
Adopting healthy lifestyle	915778.500	2067181.500	–13.814	<.001
Coping with day-to-day stress	898958.000	2050361.000	–14.424	<.001
Improving workplace productivity	908140.500	2059543.500	–14.016	<.001
Staying healthy during the COVID-19 pandemic	896486.500	2047889.500	–15.060	<.001

**Table 3:** Effect of yoga on health-related quality of life.

Health-related quality of life characteristic and category		Regular yoga practitioners,^a^ n (%)	Nonregular yoga practitioners,^b^ n (%)	*P* value^c^	
**Ability to relax**					<.001
	Applies to me very much	789 (47.91)	478 (31.51)		
	Applies to me to a considerable degree	391 (23.74)	600 (39.56)		
	Applies to me to some degree	212 (12.87)	258 (17)		
	Does not apply to me	255 (15.48)	181 (11.93)		
**Frequency of nervousness and stress**					<.001
	Applies to me very much	98 (5.95)	94 (6.19)		
	Applies to me to a considerable degree	172 (10.44)	212 (13.97)		
	Applies to me to some degree	650 (39.47)	561 (36.99)		
	Does not apply to me	727 (44.14)	650 (42.85)		

^a^N=1647.

^b^N=1517.

^c^Chi-square test *P*<.05, *df*=3.

### Effect of Heartfulness Meditation on HRQOL

Notably, regular Heartfulness meditation practitioners reported a higher statistically significant impact on all HRQOL domains: quality of life (*U*=993578, *P*<.001), ability to adopt healthy lifestyle (*U*=1012703.500, *P*<.001), coping with day-to-day stress (*U*=984983, *P*<.001), improving work productivity (*U*=998981.500, *P*<.001), staying healthy during the COVID-19 pandemic (*U*=995166.500, *P*<.001; Table 4). Further, the Heartfulness meditation practice group had a greater ability to relax (*df*=3, *P*<.001) and experienced a lower frequency of nervousness and stress (*df*=3, *P*<.001) as compared to the non–Heartfulness meditation group (Table 5).

**Table 4:** Effect of Heartfulness meditation on health-related quality of life.

Health-related quality of life characteristic	Mann-Whitney *U* test	Wilcoxon W	Z statistic	*P* value
Quality of life	993578.000	1611294.000	–6.329	<.001
Adopting healthy lifestyle	1012703.500	1630419.500	–5.538	<.001
Coping with day-to-day stress	984983.000	1602699.000	–6.700	<.001
Improving workplace productivity	998981.500	1616697.500	–6.083	<.001
Staying healthy during the COVID-19 pandemic	995166.500	1612882.500	–6.491	<.001

**Table 5:** Effect of Heartfulness meditation on health-related quality of life.

Health-related quality of life characteristic and category			Heartfulness meditation group,^a^ n (%)		Non–Heartfulness meditation group,^b^ n (%)		*P* value^c^	
**Ability to relax**								<.001
	Applies to me very much	866 (42.18)		401 (36.09)				
	Applies to me to a considerable degree	572 (27.86)		418 (37.62)				
	Applies to me to some degree	303 (14.76)		170 (15.31)				
	Does not apply to me	312 (15.20)		122 (10.98)				
**Frequency of nervousness and stress**								<.001
	Applies to me very much	94 (4.58)		69 (6.21)				
	Applies to me to a considerable degree	195 (9.5)		131 (11.79)				
	Applies to me to some degree	807 (39.31)		488 (43.93)				
	Does not apply to me	957 (46.61)		423 (38.07)				

^a^N=2053.

^b^N=1111.

^c^Chi-square test *P*<.05, *df*=3.

### Effect of Years of Heartfulness Meditation Practice on HRQOL

Participants were categorized in three groups based on their response to the number of years of experience with Heartfulness meditation practice: (1) not currently practicing but had previously tried the form of meditation, (2) ≤1 year, and (3) ≥2 years of meditation practice. A total of 1217 participants (59.28%) reported ≥2 years of Heartfulness meditation experience, 786 (38.28%) reported ≤1 year of Heartfulness meditation experience, and 50 (2.44%) reported having previously tried this form of meditation but not currently practicing it.

Significant differences within the groups were observed through a Kruskal-Wallis test. The test indicated that quality of life (*H*=77.33, *P*<.001), adopting a healthy lifestyle (*H*=55.54, *P*<.001), coping with day-to-day stress (*H*=61.78, *P*<.001), improving workplace productivity (*H*=67.64, *P*<.001), and staying healthy during the COVID-19 pandemic (*H*=64.79, *P*<.001) differed between at least one group within the Heartfulness meditation practice group. Post hoc analysis revealed that there was a higher HRQOL for all domains observed for participants with ≥2 years of meditation practice as compared to the other two groups (*P*<.001; Table 6).

**Table 6:** Post hoc analysis within the Heartfulness meditation practice group.

Health-related quality of life domain and comparison between groups with different years of Heartfulness meditation experience		Mean difference (group 1 – group 2)	SE	*P* value^a^	Confidence interval	
					Lower bound	Upper bound
**Quality of life**						
	Not currently practicing vs <1 year	–.38682	.23217	.10	–.8421	.0685
	Not currently practicing vs ≥2 years	–.56687	.22970	*.01*	–1.0173	–.1164
	<1 year vs not currently practicing	.38682	.23217	.10	–.0685	.8421
	<1 year vs ≥2 years	–.18005	.07284	*.01*	–.3229	–.0372
	≥2 years vs not currently practicing	.56687	.22970	*.01*	.1164	1.0173
	≥2 years vs <1 year	.18005	.07284	*.01*	.0372	.3229
**Adopting healthy lifestyle**						
	Not currently practicing vs <1 year	–.40880	.23458	.08	–.8688	.0512
	Not currently practicing vs ≥2 years	–.55673	.23208	.*02*	–1.0119	–.1016
	<1 year vs not currently practicing	.40880	.23458	.08	–.0512	.8688
	<1 year vs ≥2 years	–.14793	.07360	*.04*	–.2923	–.0036
	≥2 years vs not currently practicing	.55673	.23208	*.02*	.1016	1.0119
	>2 years vs <1 year	.14793	.07360	*.04*	.0036	.2923
**Coping with day-to-day stress**						
	Not currently practicing vs <1 year	–.44081	.23341	.06	–.8986	.0169
	Not currently practicing vs ≥2 years	–.59947	.23092	*.009*	–1.0523	–.1466
	<1 year vs not currently practicing	.44081	.44081	.06	–.0169	.8986
	<1 year vs ≥2 years	–.15866	.07323	*.03*	–.3023	–.0150
	≥2 years vs not currently practicing	.59947	.23092	*.009*	.1466	1.0523
	≥2 years vs <1 year	.15866	.07323	*.03*	.0150	.3023
**Workplace productivity**						
	Not currently practicing vs <1 year	–.32921	.23048	.15	–.7812	.1228
	Not currently practicing vs ≥2 years	–.60412	.22803	*.008*	–1.0513	–.1569
	<1 year vs not currently practicing	.32921	.23048	.15	–.1228	.7812
	<1 year vs ≥2 years	–.27491	.07231	*<.001*	–.4167	–.1331
	≥2 years vs not currently practicing	.60412	.22803	*.008*	.1569	1.0513
	≥2 years vs <1 year	.27491	.07231	*<.001*	.1331	.4167
**Staying healthy during the COVID-19 pandemic**						
	Not currently practicing vs <1 year	–.50092	.21232	*.02*	–.9173	–.0845
	Not currently practicing vs ≥2 years	–.71803	.21006	*.001*	–1.1300	–.3061
	<1 year vs not currently practicing	.50092	.21232	*.02*	.0845	.9173
	<1 year vs ≥2 years	–.21711	.06662	*.001*	–.3478	–.0865
	≥2 years vs not currently practicing	.71803	.21006	*.001*	.3061	1.1300
	≥2 years vs <1 year	.21711	.06662	*.001*	.0865	.3478

^a^Values in italics are statistically significant.

## Discussion

### Principal Findings

Overall, this study showed that a regular yoga and meditation practice was associated with factors promoting health and well-being, with long-term meditation practice associated with increased benefits. This study is one of the first cross-sectional studies to analyze the effects of a 100-day virtual yoga and meditation program and has 3 key findings. First, the demographic results suggest most practitioners are female and students/educated. Our findings corroborate those of other studies in several countries such as the United Kingdom, United States, and Australia, where yoga and meditation practitioners were mostly female and educated [35-38]. Although those studies reported an average age between 39-41 years, this study, in contrast, had a younger population with an average age of 33.8 years and mostly student participants. These findings concur with recent studies in the Indian setting reporting that students and a younger population make up most participants for yoga events [39,40]. Recent literature has reported an increased interest among young people in India to incorporate yoga as part of their fitness regimen [40]. Nevertheless, we speculate the larger participation from India in this global event, as compared to other countries, is because of the broader presence of the event organizers (Heartfulness Institute, CCRYN, Ministry of Ayush, SVYASA Yoga University, and Patanjali Yoga Institute) in India.

Second, participants were highly engaged throughout the program period, given that 100 days of yoga and meditation is a substantial time commitment. A notable average participation rate of 71 days (SD 32), with 53.22% attending sessions every day for 100 days, suggests that participants were willing to engage in an online activity to enhance their well-being. Limited research exists to compare engagement rates of programs centered around International Yoga Day events with previous literature [39,40].

Third, results examining the effect of yoga demonstrated that regular practice had a statistically significant positive impact on all 7 domains of HRQOL. Similar results have been reported by several studies examining the effect of yoga on mental and physical health [40]. There is overwhelming evidence indicating that the frequency of yoga practice positively predicts its health benefits [35,36,38,41,42]. Another important finding of this study was that meditation had a statistically significant positive impact on all the HRQOL domains (*P*<.05). Interestingly, participants with ≥2 years of experience reported a higher impact on all domains of HRQOL as compared to participants with ≤1 year of meditation practice. The findings imply that sustained practice may cumulatively increase the benefits for well-being. This contrasts with a recent study that found no association between years of meditation practice and mental well-being [42]. Nevertheless, findings from this study concur with previous literature suggesting a positive correlation between perception of health, well-being, and years of meditation practice [43-45].

### Limitations

Although this study provided new evidence about characteristics of yoga and meditation practitioners in a 100-day virtual yoga and meditation program, there were several limitations. The data were cross-sectional in nature; therefore, causality cannot be inferred. A priority for future research includes using longitudinal designs to examine the causal relationship between meditation practice and key outcomes of interest of the study. Moreover, all participants included in the evaluation self-selected to participate in the program. This may have contributed to a potential inclusion bias of those with an interest in yoga and meditation. Further, 100 days of yoga is a substantial time commitment and such a program may have limited active participation from a broader population (eg, full-time employees). Additionally, there was an uneven distribution of members from participating countries and findings of the study may be generalizable only to Heartfulness meditation practitioners.

### Conclusion

This is one of the first cross-sectional studies to analyze the effects of a 100-day virtual yoga and meditation program. Overall, a yoga and meditation practice was found to be an effective tool to promote HRQOL. Regular yoga and meditation practice was associated with factors promoting health and well-being, with long-term meditation practice associated with increased benefits.

## Abbreviations

CCRYN: Central Council for Research in Yoga and Naturopathy
CHERRIES: Checklist for Reporting Results of Internet E-Surveys
